# Whole-Genome Sequence Insight into the Plant-Growth-Promoting Bacterium *Priestia filamentosa* Strain AZC66 Obtained from *Zygophyllum coccineum* Rhizosphere

**DOI:** 10.3390/plants12101944

**Published:** 2023-05-10

**Authors:** Ashraf Khalifa, Noorah Alsowayeh

**Affiliations:** 1 Biological Sciences Department, College of Science, King Faisal University, Al-Ahsa 31982, Saudi Arabia; 2 Botany and Microbiology Department, Faculty of Science, Beni-Suef University, Beni-Suef 62511, Egypt; 3 Department of Biology, College of Education (Majmaah), Majmaah University, Al-Majmaah 11952, Saudi Arabia; noalsowayeh@mau.edu.sa

**Keywords:** cowpea, genome, plant biostimulation, *Priestia*, rhizosphere

## Abstract

This study aimed to isolate, screen the plant-growth-enhancing features, and explore the whole-genome sequence of AZC66 isolated from the rhizosphere of *Zygophyllum coccineum* and determine its biostimulating effects on the growth of cowpea under greenhouse conditions. Salkowski reagent was used to measure AZC66’s indole acetic acid production. AZC66’s inorganic phosphate solubility on Pikovskaya agar was evaluated using tricalcium phosphate. The results indicated the ability of AZC66 to fix nitrogen, produce IAA (66.33 ± 0.44 μg mL^−1^), solubilize inorganic phosphate, and exhibit the activity of ACC deaminase (278.40 ± 21 mol -ketobutyrate mg^−1^ h^−1^). Cowpea’s root and shoot dry weights were also significantly increased after in vitro inoculation with AZC66. The identity of AZC66 was confirmed as *Priestia filamentosa*, and 4840 genes were predicted in its genome. The gene sequences were compared against the Kyoto Encyclopedia of Genes and Genomes (KEGG) database, and the results showed that the top three pathways wherein the maximum number of genes are involved are signaling and cellular processes, genetic information processing, and carbohydrate metabolism. The genome sequencing of the strain AZC66 revealed a number of genes implicated in plant biostimulation activities such as nitrogen fixation (*nifU*), phytohormone synthesis (*trpAB* genes), phosphate solubilization (*PhbCEF*, *pstABCS*, and *phoU*), and siderophore formation (*FbpA*, *feoAB*, and *fetB*). The AZC66 genome contained numerous genes involved in nitrogen metabolism, nitrogen regulation, and the nitrate reduction pathway. The phenazine biosynthetic gene in AZC66 demonstrated biocontrol and soil survival properties. The trehalose synthesis genes in AZC66 may help plants resist osmotic and salt stress. The discovery of glycine betaine, cold shock, and heat shock protein genes demonstrated that AZC66 could withstand harsh conditions. AZC66 might be used to create robust, sustainable biological fertilizers for future agricultural use in Saudi Arabia. Furthermore, the predicted adaptable metabolic pathways might serve as the basis for potential biotechnological applications in agriculture and industry.

## 1. Introduction

A variety of microbial groups grow and interact with roots in the rhizosphere due to the presence of nutrients secreted by plant roots. Root exudates are a group of soluble organic substances synthesized and released by roots, including amino acids, sugars, growth factors, and enzymes [1,2,3]. These nutrients shape the abundance and composition of microbes around plant roots. The number of microorganisms in rhizospheric soil is significantly higher than in bulk soil [4]. Plant-growth-promoting rhizobacteria (PGPR) are advantageous plant probiotics that boost plant development both directly and indirectly, including with respect to iron sequestration, plant hormone production, nitrogen fixation and inorganic phosphate solubilization, and anti-phytopathogen and anti-stressor production [2].

*Zygophyllum coccineum* is a perennial herb in the family of flowering plants designated *Zygophyllaceae*. It grows in many places in Saudi Arabia, such as the strip of Al-coastal Uqair [5]. *Bacillus*, *Enterobacter*, and *Janibacter*, which help plants grow, have been found in *Zygophyllum* spp. [6]. These beneficial bacteria have displayed remarkable activity with respect to ameliorating the negative impacts of salinity stress on wheat [6], highlighting their potential role in sustainable agriculture. Among other important crops, the cowpea (*Vigna unguiculata*), also called black-eyed pea, is an annual herbaceous grain legume that is grown in many places around the world, including Saudi Arabia [7]. People eat cowpea grains because they are rich in proteins, vitamins, and minerals. The whole plant is commonly used as animal forage. Furthermore, the cowpea is characterized by its ability to grow in harsh conditions, prevailing in semiarid regions where the plant exhibits a tolerance for sandy soil and low rainfall. Additionally, the roots of the cowpea establish symbiotic associations with nitrogen-fixing rhizobia that develop the plant’s root nodules [8]. This trait renders the cowpea a valuable crop for resource-limited farmers, for which it is well-suited to inter-cropping. Cowpea plants occupy over 14.4 million ha of agricultural land worldwide and yield an annual production value of 8.9 million tonnes [7], implying their global economic and food-related significance. The use of PGPR could be a promising strategy for maintaining cowpea health and yield.

*Priestia* is a genus of mostly Gram-positive rod-shaped bacteria in the *Bacillaceae* family of Bacillales. *Priestia* species were previously known as *Bacillus* species. Based on robust comprehensive phylogenomic and comparative analyses of *Bacillus* species, several new genera, such as *Priestia* and *Brevibacillus*, have been proposed based on the presence of unique conserved signature indels for each of the identified clades [9]. The *Priestia* name honors the British microbiologist Fergus G. Priest for his substantial efforts concerning *Bacillus* systematics. *Priestia* contains 10 validly published species, including the recently described species *P. veravalensis* [10] (https://lpsn.dsmz.de/genus/Priestia, accessed on 5 March 2023). *Priestia* spp. have significant beneficial roles in various plant species that are enacted via diverse mechanisms [11,12,13,14]. For example, *P. megaterium* that was isolated from the halophytic plant species *Bolboschoenus planiculmis* induced a significant enhancement in the growth parameters of *Arabidopsis thaliana* and *Brassica rapa* under salt and drought stresses [12]. Additionally, *P. endophytica* that was obtained from fenugreek rhizospheric soil (*Trigonella foenum-graecum*) promoted the growth of the same plant species [13]. Furthermore, *P. filamentosa* and *P. aryabhattai* boosted cowpea growth under drought and nutrient deficiency and altered the root transcriptome [14]. The bacterial inoculation of economically important plants such as cowpea may significantly aid efforts to close the gap between production and consumption, which is important for food security in the long term.

The whole-genome sequencing of *Priestia* species has been widely publicized and indicated this species’ valuable potential uses, including its traits that help plants grow. For example, the genome of the plant-growth-promoting bacterium *Priestia* sp. strain TSO9, which is associated with *Triticum turgidum* subsp. *durum* [15] and *P. megaterium*, had recently been revealed [16]. Deciphering the whole-genome sequences of such a bacterial strain will surely pave the way for various agricultural and industrial applications in the future. Nevertheless, there are not many reports regarding PGPR isolated from *Z*. *coccineum*, which suggests that more research is needed to explore the beneficial features of the bacteria associated with the rhizosphere of this plant. Therefore, this study aimed at isolating and screening the plant-growth-enhancing features and whole-genome sequence of AZC66 isolated from the rhizosphere of Z. *coccineum* and to determine its biostimulating effects on the growth of cowpea under greenhouse conditions.

## 2. Materials and Methods

### 2.1. Soil Collection and Bacterial Isolation

On 28 March 2019, one rhizospheric soil sample of a Z. *coccineum* plant was collected from Al-Uqair region (25°37′35.60″ N, 49°54′59.19″ E). The plant was uprooted along with the surrounding soil. In a lab, 99 mL of 0.85% NaCl was added to 1 g of soil, incubated (with shaking applied at 150 rpm) at 30 °C for 30 min, and then serially diluted. On nutrient agar plates, aliquots (100 µL) were streaked and incubated at 30 °C for 48–72 h. Distinctive single colonies were selected after incubation, re-streaked on NA plates, and then kept at −80 °C in 15% glycerol. AZC66’s colonial characteristics were then noted.

### 2.2. Evaluation of Plant-Growth-Stimulating Features

The AZC66 strain’s production of indole acetic acid was evaluated using Salkowski reagent [17]. On Pikovskaya agar, the AZC66 strain’s capacity to solubilize inorganic phosphate was tested using tricalcium phosphate (Ca_3_(PO_4_)_2_) as the P source, and the development of transparent zones around bacterial colonies was taken as a positive sign for this trait after a 5-day incubation period at 30 °C [18]. AZC66’s AAC deaminase activity was evaluated according to the method mentioned earlier [19], for which the standard curve of α-ketobutyrate was used.

### 2.3. Cross-Inoculation Study

Cross-inoculation of cowpea seed grains (varieties of Lupia Baladi) was performed in a lab to determine whether AZC66 could help plants grow [20]. Briefly, healthy seeds were surface-sterilized via submergence in 70% ethanol for 10 s and then in 3% H_2_O_2_ for 3 min. Seeds were then rinsed several times with sterilized distilled water. Under aseptic conditions, the seeds were inoculated for 24 h with an active AZC66 suspension (5 × 10^6^ CFU mL^−1^). Bacterial growth was carried out in nutrient broth maintained at a temperature of 30 °C for 24 h. The inoculated seeds were grown in plastic pots (five seeds/pot) with 0.5 kg of sterilized soil (clay: sand ratio of 1:1) in a growing room at 25 °C with 50% relative humidity and a 14 h photoperiod. Treatments were performed in triplicate, and a negative control with no added bacteria was incorporated. After 2 weeks, the length and dry weight of roots and shoots were measured.

### 2.4. Statistical Analysis

The data are represented as the mean of three replicates and the standard deviation (SD). The data were processed using analysis of variance (ANOVA), and mean comparisons were performed in Microsoft Excel using a post hoc test. Differences in means were regarded as statistically significant at a *p* value less than 0.05.

### 2.5. DNA Isolation and Qualitative and Quantitative Analysis

Cells were collected from the AZC66 culture that had been actively growing in nutrient broth after 18 h of incubation at 30 °C and centrifugation at 7000 rpm for 15 min. The total genomic DNA of the cells was then extracted using the Xcelgen Bacterial DNA Isolation Kit. A total of 3 μL was deposited on a 0.8% agarose gel in order to check the quality of the DNA for a single intact band. The DNA was analyzed on gel for 30 min at 110 V. The Qubit^®^ 2.0 Fluorometer (Life Technologies, Singapore) was used to determine the concentration of 1 µL of each sample. Preparation of the library: A paired-end sequencing library was constructed with the help of the NEBNext Ultra DNA Library Prep Kit for Illumina. Employing 200 ng of DNA, the library preparation process was initiated. Covaris mechanically sheared the DNA into smaller fragments, which was followed by a phase of end-repair in which an ‘A’ is appended to the 3’ ends, thus preparing the DNA fragments for adapter ligation. The platform-specific adapters were then ligated to both ends of the DNA fragments. These adapters contain the sequences necessary for binding dual-barcoded libraries to a flow cell for sequencing, thereby allowing for the PCR amplification of adapter-ligated fragments and the attachment of Illumina sequencing primers. Using HiFi PCR Master Mix, a high-fidelity amplification phase was carried out to assure optimum yields from limited quantities of starting material.

The quantity and quality (QC) of the library were checked using a Bioanalyzer. According to the manufacturer’s instructions, the amplified library was analyzed on a Bioanalyzer 2100 (Agilent Technologies) using a High Sensitivity (HS) DNA chip.

### 2.6. Bioinformatics Analysis

Cluster generation and sequencing: Once the library’s Qubit concentration and the average peak size from the bioanalyzer profile were obtained, the library was moved to the Illumina platform for cluster generation and sequencing. Paired-end sequencing enabled the sequencing of template fragments in both forward and reverse directions. De novo assembly: Velvet v1.2.10 was used to construct high-quality PE reads of the bacterial strain from scratch, and the assembly was optimized at Kmer-123. The scaffolds were further gap-filled (stretch of ‘Ns’ were filled with A, T, G, and C bases) using PE read information via GapCloser v1.12 software. Gene prediction was followed by annotation procedures. Identification of tRNAs, rRNAs, and CDS was carried out using tRNAscan-SE (version 2.0), RNAmmer (version 1.2), and Prodigal (version 2.60), respectively. A similarity search against NR, Uniprot, COG, and Pfam was performed using BLASTP (version 2.2.30). The predicted proteins’ KEGG pathways were assigned using the KEGG server (KAAS). Plant biostimulation traits, pathogen suppression, abiotic stress tolerance, rhizosphere competence, carbohydrate metabolism, and other important, relevant functions were identified among the annotated genes.

SSR (Simple Sequence Repeats) identification: SSRs were identified in the scaffold sequences of each sample using the MISA perl script. The criteria that were used for SSR identification are as follows: (1) a di-nucleotide pattern should appear at least six times; (2) a tri-nucleotide pattern should appear ≥five times; (3) a tetra-nucleotide pattern should appear ≥five times; (4) a penta-nucleotide pattern should appear ≥five times; and (6) a hexa-nucleotide pattern should appear ≥five times.

Gene prediction: The 31 scaffolds obtained were subjected to gene prediction using Prodigal v2.60, which resulted in the identification of 4840 coding sequences.

Functional annotation of predicted genes: The predicted 4840 proteins encoded by genes were subjected to a similarity search against NCBI’s non-redundant (NR) database using the BLASTP algorithm with an e-value threshold of 8 × 10^5^. The statistics of blast annotation are shown in the following table.

Distribution of GO sequences: GO annotation was derived for NR-annotated proteins using the blast2GO command-line version 1.4.1. GO sequence distributions aid in the specification of all annotated nodes constituting GO functional groups. Using the KEGG automatic annotation server, pathway analysis, ortholog assignment, and mapping of genes to biological pathways were performed. (KAAS). All 4840 gene sequences were compared to the KEGG database using BLASTP with a bit-score threshold of 60 (default). Generation of circular chromosome map:

The Proksee (https://proksee.ca, accessed on 6 December 2022) server was used to construct a circular chromosome map of *Priestia filamentosa* strain AZC66, which included the CDS, tRNAs, rRNAs, and guanine–cytosine (GC) bias content of the bacterial strain.

Database accession number and data availability: The genome sequence database of the AZC66 strain, as described in the manuscript, has been deposited in the NCBI SRA module and is accessible via Accession No. SUB12144779 (PRJNA890665).

## 3. Results

Out of 30 morphologically different bacterial isolates, AZC66 was chosen to have its whole-genome re-sequenced. AZC66 showed promise with respect to stimulating plant growth during the initial screening. AZC66 produced smooth, round colonies with a complete edge (Table 1). 

### 3.1. Plant-Biostimulating Traits of the Strain AZC66

Exactly 66.33 ± 0.44 μg mL^−1^ of IAA, a plant-growth-promoting hormone, was produced by AZC66 when the growth medium was supplemented with tryptophan (1 mg mL^−1^). The qualitative evaluation of AZC66’s ability to solubilize inorganic phosphate generated the results shown in Table 1. A halo zone formed around the AZC66 colonies grown on Pikovskaya agar with calcium triphosphate added. This observation demonstrates that AZC66 has a remarkable capacity for phosphate solubility. In addition, AZC66 exhibited an ability to grow well in Jensen’s medium. (Table 1). The cowpea seedlings that were inoculated in vitro with AZC66 exhibited markedly improved growth characteristics. The lengths of the treated cowpea plants’ roots and stems significantly increased from 3.5 ± 0.66 cm to 7.9 ± 0.35 cm and 6.3 ± 0.42 cm to 9.8 ± 0.22 cm, respectively, when compared to the untreated plants (Table 2). Due to the plants’ inoculation with AZC66, the dry weights of the plant’s roots and shoots increased from 19.9 ± 0.73 mg to 38.3 ± 0.69 mg and 36.3 ± 0.55 mg to 75.1 ± 0.35 mg, respectively (Table 2). 

### 3.2. Sequence, Assembly, and Gene Prediction of the Entire Genome

**Figure 1:**
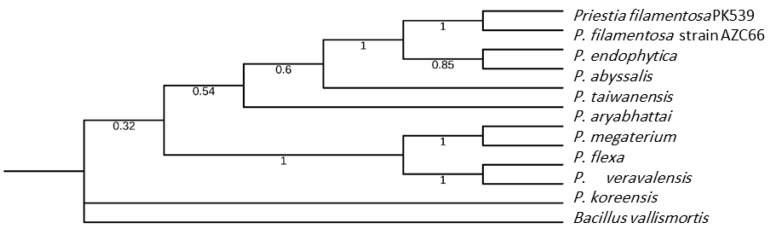
Phylogenetic relationships between *Priestia filamentosa* strain AZC66 and closely related species (based on the 16S rRNA gene): *P. megaterium* (BioProject: PRJNA390877); *P. aryabhattai* (BioProject: PRJNA414277); *P. flexa* (BioProject: PRJNA647851), *P. abyssalis* (BioProject: PRJNA350189), *P. endophytica* (BioProject: PRJNA317263), *P. koreensis* (BioProject: PRJNA293250), *P. taiwanensis* (BioProject: PRJDB10509) and *Priestia veravalensis* (BioProject: PRJNA302099). *B. vallismortis* (BioProject: PRJNA412146) was used as an outgroup. Using MEGA11, the maximum likelihood method and the JTT matrix-based model were employed to build this relationship (based on 1000 bootstrap replications). The percentage of the 500 replicated trees in which the same taxa grouped together in the bootstrap test is shown next to each branch.

When sequencing the genome of this bacterial strain with the Illumina HiSeq 2500 sequencing platform, a total of 24,068,502 processed reads were obtained for AZC66 (Table 3). The GC percentage was 36.42 percent. AZC66’s genome was ultimately assembled in 31 scaffolds with a total length of 4,893,880 nucleotides, including gaps (‘Ns’) (as depicted in Table 3, in which 4,893,873 bp are shown). The scaffolds’ N50 was 1,051,362 base pairs, and the average length was 157,866.93 base pairs. The AZC66 strain had a circular chromosome with 4.89 Mb base pairs and an average G + C content of 36.42% (genome; Table 3). The phylogenetic relationships between *Priestia filamentosa* strain AZC66 and closely related species based on the 16S rRNA gene are presented in Figure 1. One can notice that AZC66 was clearly clustered with the recognized *P. filamentosa* strain *PK539.*

**Figure 2:**
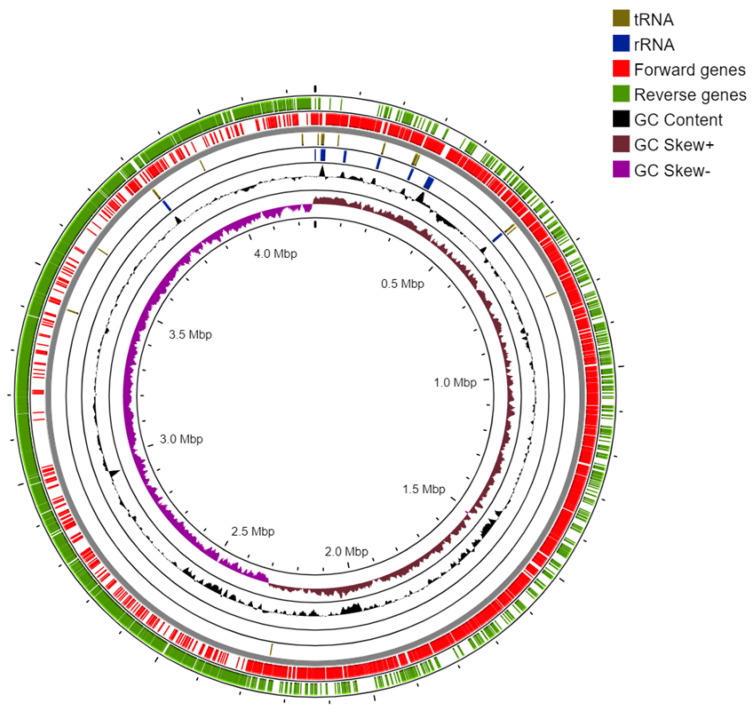
A circular chromosome map of *Priestia filamentosa* strain AZC66 created using Proksee Server. This map shows the spread of CDS, tRNAs, and rRNAs, and the GC content shift.

Resultantly, 4840 genes, 9 rRNA genes, and 75 tRNA genes were obtained (Table 1). Around 4815 protein-coding genes with predicted functions and 25 genes without predicted functions were identified. Based on the sequence results, the size of the genome was determined to be 4.89 Mb. Phylogenetic analysis confirmed that this strain of bacteria belongs to the *Bacillaceae* family. The circular chromosome map of *P. filamentosa* strain AZC66, which involves the distribution of CDS, tRNAs, and rRNAs and the GC content skew (generated using Proksee Server), is illustrated in Figure 2. 

AZC66’s putative microsatellites were identified using MISA script, revealing that the trinucleotide repeats were the most abundant, followed by dinucleotide repeats. Considering the flanking region of 150 bp, a total of 10 sequences flanking SSRs were provided that can be used for further validation through PCR. A total of 4840 genes were predicted from 31 scaffolds using Prodigal software. The GO distribution was determined using the Blast2GO command line tool. A total of 2794 genes were given GO terms. Of those, 1960, 1388, and 2169 were assigned to the biological process category, the cellular component category, and the molecular function category, respectively. All 4840 gene sequences were compared against the Kyoto Encyclopedia of Genes and Genomes (KEGG) database using BLASTP with a threshold bit-score value of 60. The results show that the top three pathways with the highest numbers of genes involved are signaling and cellular processes, genetic information processing, and carbohydrate metabolism.

Non-coding RNA prediction: The predicted total number of tRNAs was 75, 74 of which were tRNAs decoding the standard 20 AAs and 1 was a tRNA with an undetermined/unknown isotype (Table 3). For the bacterial strain AZC66, eight 5S rRNA and one 16S rRNA were identified.

Simple Sequence Repeats (SSR): SSRs or microsatellites are paired copies of 2 to 6 bp nucleotide patterns that are very variable and found in all known genomes. Therfore, the MISA perl script was used to find SSRs in the scaffold sequences. An in-house Python script was used to find SSRs flanked by 150 bp both upstream and downstream. This script can be used to build primers. Gene prediction: The 31 scaffolds obtained were subjected to gene prediction using Prodigal v2.60, which resulted in the identification of 4840 coding sequences. Further distribution of genes was performed according to their length. The majority of genes ranged in length from 1000 bp to 5000 bp.

### 3.3. Functional Annotation of Predicted Genes

The predicted 4840 proteins of genes were subjected to a similarity search against NCBI’s non-redundant (nr) database using the BLASTP algorithm with an e-value threshold of 8 × 10^5^. The statistics regarding blast annotation are shown in Table 4. The total number of proteins was 4840, the No. of proteins with hits was 4815, and the No. of proteins with no hits was 25.

### 3.4. Distribution of Gene Ontology Sequences

**Figure 3:**
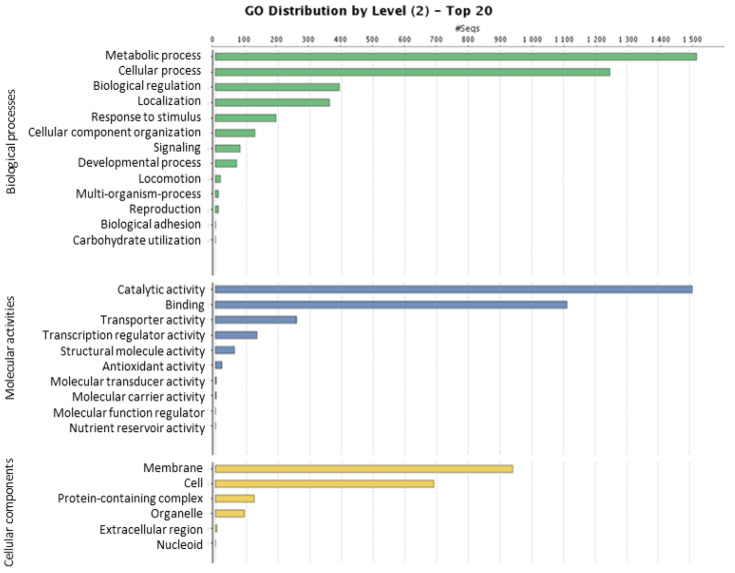
Distribution of Gene Ontology (GO) Sequences of the strain AZC66; GO annotation for proteins marked in the NR database was retrieved using the blast2GO command-line version 1.4.1. All the annotated nodes that make up a GO functional group may be more precisely described with the aid of GO sequence distributions. The GO functional grouping system classifies genes with comparable roles into the same category. The GO sequence patterns of all three GO domains—biological processes, molecular activities, and cellular components—were analyzed.

The blast2GO command line version 1.4.1 was used to retrieve GO annotation for proteins annotated in the NR database. All the annotated nodes that make up a GO functional group may be more precisely described with the aid of GO sequence distributions. The GO functional grouping system classifies genes with comparable roles into the same category. All three GO domains—biological processes, molecular functions, and cellular components—had their GO sequence distributions analyzed (Figure 3). GO words were assigned a total of 2794 genes. In total, there were 1960 processes, 1388 cellular components, and 2169 organisms. 

*Pathway Analysis*: The KEGG automated annotation service was used for pathway analysis, ortholog assignment, and the mapping of genes to biological pathways. (KAAS). Using BLASTP and a bit-score cutoff of 60, all 4840 gene sequences were compared to the KEGG database (default). Carbohydrates, lipids, nucleotides, amino acids, glycans, cofactors, vitamins, terpenoids, polyketides, and other key biomolecules were all represented by the mapped proteins. The proteins corresponded to genes that regulate biological functions, the environment, and information processing inside the genome. Table 4 displays the distribution of genes according to their respective pathway assignments.

## 4. Discussion

Now more than ever, it is clear that the beneficial effects of bacteria are indispensable for sustainable agriculture, as they can minimize the negative environmental and human-health-related impacts induced by the use of chemical fertilizers. Searching for efficient bacterial strains with plant probiotic traits is of paramount importance for sustainability. One approach in this regard is to explore the rhizobacteria associated with wild plants such as *Zygophyllum coccineum* growing under severe climatic conditions, e.g., under elevated temperatures, drought, and salinity. Thus, the current study reported the isolation of *P. filamentosa* AZC66 PGPR from *Z. coccineum* rhizosphere. The strain had a rod-shaped morphology and tested positive with respect to Gram staining and endospore production. These features are identical to those reported for *Bacillus filamentosus* [21] and *P. filamentosus* [9]. Additionally, *P. filamentosa* AZC66 exhibited a number of plant-biostimulating features, including phosphate solubilization, ACC deaminase activity, and the production of IAA. Plants need phosphorus (P). In soil, phosphorus exists in forms that are biologically inaccessible for plants. Phosphate-solubilizing bacteria play an important role in the availability of phosphorus to plants by secreting phosphatases and organic acids. These bacteria have also been shown to solubilize phosphate [22,23]. PGP helps plant roots absorb phosphorus for growth and productivity. IAA increases ACC production via ACC synthetase [24], and AZC66 increases plant growth via IAA production. AZC66’s growth on nitrogen-free Jensen’s medium may suggest nitrogen fixation [25,26]. Nitrogenase, a complex enzyme, fixes nitrogen. Many studies have confirmed the nitrogen-fixing ability of *Bacillus* spp [27]. PGPR fixes nitrogen to meet plant nitrogen needs, thereby improving soil fertility and plant yields. AZC66 had higher ACC deaminase activity (278.40 ± 21.18 μmol α-ketobutyrate mg^−1^ h^−1^) than *P. aryabhattai* from the leaf and stem parts of *Triticum aestivum* L. (wheat) (23.2 (278.40 ± 21.18 μmol α-ketobutyrate mg^1^ h^−1^)) [28] but lower activity than *Aneurinibacillus aneurinilyticus* and *Paenibacillus* sp. (1677 nmol α-ketobutyrate mg protein^−1^ h^−1^) followed by ACC06 (1589 nmol α-ketobutyrate mg protein^−1^ h^−1^) [28]. These findings indicate the variability in the production of ACC deaminase among bacterial strains. Further confirmation of the biostimulating capacity of ACZ66 was obtained from the in vitro inoculation of cowpea with said bacterial strain. AZC66-inoculated cowpea plants grew better in vitro (i.e., the root and stem lengths and dry weights of the roots and shoots had increased). Previous studies showed that *Priestia* spp. have substantial beneficial functions in a variety of plant species that are enacted in a variety of ways [11,12,13,14]. For example, *Priestia megaterium*, isolated from the halophytic plant species *Bolboschoenus planiculmis*, significantly improved the growth characteristics of *Arabidopsis thaliana* and *Brassica rapa* under salt stress and drought conditions [12]. In addition, *P. endophytica* from fenugreek rhizospheric soil (*Trigonella foenum-graecum*) encouraged the growth of the same plant species [13]. Additionally, *P. filamentosa* and *P. aryabhattai* increased cowpea growth in drought and nutrient-deficient conditions and altered the root transcriptome and genome [14]. The bacterial inoculation of economically important plants such as cowpea has the potential to significantly reduce the gap between output and consumption, which is critical for long-term food security.

The 4840 proteins predicted to be encoded by genes were subjected to a similarity search against NCBI’s non-redundant (NR) database using the BLASTP algorithm with an e-value threshold of 8 × 10^5^ There were 4840 total proteins, 4815 proteins with hits, and 25 proteins without any results. The top-hits species distribution revealed that the majority of strikes were against the *B. filamentosus* species. According to previously published data, the complete-genome sequences of *Bacillaceae* members have an average genome size of 4.3 Mb and 4364 protein-coding gene sequences. The reported genome sizes of members of this family range from 2.8 to 5.8 Mb [29]. It has been reported that there is a strong correlation between the extent of the genome and the number of encoding genes [30]. Even though genome size and gene number are significantly correlated in prokaryotes, in eukaryotes, the overwhelming majority of nuclear DNA is non-coding. The prediction of functional protein-coding genes (PCG) was made based on the genomic protein output after processing at NCBI. Multiple genes involved in biomolecule transport, metabolism, the synthesis of cellular components, and diverse metabolic pathways were identified in the AZC66 PCG. The AZC66 genomic sequence dataset revealed a diverse set of genes potentially involved in plant-growth-promoting activities such as nitrogen fixation, phytohormone production (IAA, cytokine), phosphate solubilization, and siderophore production. To support this finding, research into AZC66’s plant-growth-promoting traits could ensure a higher cowpea crop yield. The presence of nitrogen fixation genes such as *nifU* in the strain AZC66 provided strong evidence that this strain can fix nitrogen by converting dinitrogen gas into ammonium. Plants can easily utilize such fixed nitrogen compounds. *Bacillus* spp. have been reported to be active nitrogen fixers in chicken pea plants growing in nitrogen-deficient soil environments [31]. AZC66 contains genes for nitrogen metabolism and regulation, ammonia and urea transport, and nitrate reduction. It also contains genes such as *nifU* and *nif3*-like, which are involved in fixing nitrogen. (Table 2). Phosphorus in soil is either fixed or immobilized, thus limiting its availability in plants. Table 3 shows the presence of PCG in AZC66, which is involved in phosphonate degradation (*ispE* and *phnCE*) and phosphate transport (*pstABCS*, *phoUH*, *gltP*, and *ugpC*). Similarly, AZC66 contains iron transport and siderophore production genes such as *fbpA, feoAB*, and *fetB*. In the screened genome of strain AZC66, genes involved in phosphonate degradation and phosphate transport pathways were discovered. *PhbCEF, ispH*, *pstABCS*, *phoU, gltP*, and *ugpC* are phosphate solubilization and transport genes. This finding is supported by AZC66’s ability to solubilize inorganic phosphate compounds in Pikovskaya agar, indicating its PGP activity via this trait. *Pst* has been shown to play a role in the phosphate transport pathway in *Bacillus filamentosus* [21], *Paenibacillus polymyxa* [32], and *Bacillus subtilis* [33]. Phosphate transport genes (*pstABCS*) were unexpectedly found, although their presence is in accordance with the results of Adeleke et al. [34], who found the same genes across the genome of the plant-growth-promoting *Bacillus cereus* T4S. AZC66 contains genes for phosphonate breakdown and phosphate transport, which might accelerate phosphate utilization in host plants. Similar results have been reported with respect to *Enterobacter roggenkampii* ED5 [35] and *Pseudomonas aeruginosa* B18 [36], which are both plant-growth-promoting bacteria isolated from sugarcane root that confer biocontrol and stress tolerance properties [35,36].

Microorganisms in the soil help plants grow in areas where there is a deficiency in iron. They perform this function by producing siderophores, which make soluble iron available to plants and help them absorb it [37]. Furthermore, because microbes have a strong affinity for competing for iron in the soil, siderophore production can have lethal effects on targeted phytopathogens via ‘iron starvation’ [38]. The analyzed genome revealed the presence of iron transport genes such as *menF, entC, entB, dhbB, vibB,* and *mxcF*, indicating their involvement in siderophore production. Previously, the siderophore genes *dhbABCF* were discovered in *Bacillus* sp. strain B25, which is a biocontrol agent of the maize pathogen *Fusarium verticillioides* [39]. Rhizobacteria often produce IAA, which requires tryptophan as a starting material. The presence of aldehyde dehydrogenase and *trpAB* genes involved in L- IAA production was predicted by the genomic analysis of strain AZC66. Moreover, the presence of genes for phenazine biosynthesis, -aminobutyric acid (GABA), and D-cysteine desulfhydrase indicated their suitability for biocontrol and biodefense. The gene for phenazine biosynthesis found in AZC66 indicated its biocontrol ability in addition to its enhanced ability to survive in soil environments. Phenazines are nitrogen-containing heterocyclic compounds with redox activity that have antibacterial features and are effective against plant-pathogenic microorganisms including *Rhizoctonia* [40] and *Fusarium* [41]. Phenazine genes are present in other plant-growth-promoting bacteria including *Methylobacterium* sp. NMS14P, which is a novel bacterial strain used for the growth enhancement of maize, chili, and sugarcane [42]. These results collectively suggest that AZC66 may be useful in reducing the harmful effects of biotic and abiotic stressors on plant growth and development.

The existence of genes responsible for the synthesis of the disaccharide trehalose in AZC66 could provide an ecological advantage in terms of sustaining plant growth, particularly under osmotic and salt stressors. In support of this hypothesis, it has been suggested that trehalose generated by beneficial bacteria could activate the plant-defense system, thereby preventing drought damage [43]. Furthermore, the presence of the genes encoding highlighted the potential adaptability of AZC66 that allows it to survive in extreme conditions. Consequently, the above-mentioned traits strengthened the use of this bacterial strain as a biofertilizer, even in biotic and abiotic stress conditions, with which to improve plant growth and productivity. Deciphering the whole-genome sequences of such a bacterial strain will surely pave the way for various future agricultural and industrial applications.

## 5. Conclusions

This study analyzed the complete genome sequence of the plant-growth-promoting bacterium *P. filamentosa* strain AZC66. AZC66 was isolated from the rhizosphere of *Z. coceinium* and contained genes associated with plant growth promotion and tolerance to stress. Its genome size was comparable to that of previously reported species, and it contained over 3000 protein-coding genes with functional annotations and correlating pathways. The presence of genes and metabolic pathways in the genome analysis data amplified the performance-enhancing ability of AZC66. In the future, AZC66 could be utilized to create effective ecofriendly biofertilizers for sustainable agriculture in Saudi Arabia. Furthermore, the predicted versatile metabolic pathways could be considered the basis for potential biotechnological applications in agriculture and industry.

## Figures and Tables

**Table 1:** Morphological and plant-biostimulating features of AZC66.

Feature	AZC66
Colony shape	Circular
Color	White
Colony diameter	2–3 mm
Colony elevation	Raised
Colony margin	Entire
Gram reaction	Gram-positive rods
Nitrogen fixation	+ *
Calcium triphosphate Solubilization	+
IAA production (μg mL^−1^)	66.33 ± 0.44 μg mL^−1^
ACCD activity (nmol of α-KB mg^−1^ Pr h^−1^)	278.40 ± 21.18

*: (+) indicates positive results. ACCD: 1-Aminocyclopropane-1-Carboxylate Deaminase; α-K: α-ketobutyrate.

**Table 2:** Positive impacts of AZC66 on growth parameters of cowpea.

Feature	Control	AZC66
Root length (cm)	3.5 ± 0.66	7.9 * ± 0.35
Shoot length (cm)	6.3 ± 0.42	9.8 * ± 0.22
Root dry weight (mg)	19.9 ± 0.73	38.3 * ± 0.69
Shoot dry weight (mg)	36.3 ± 0.55	75.1 * ± 0.35

* Indicates a significant effect (*p* value < 0.05).

**Table 3:** Genomic characteristics of *Priestia filamentosa* strain AZC66.

Feature	Chromosome
Size (total gene length) (bp)	4,102,182
G + C content (%)	36.42%
Number of genes	4840
Gene density (genes per kb)	1.094
Average gene length (in bp)	847
Maximum gene length (in bp)	12,231
tRNA	75
rRNA	9
Number of genes with assigned function	4815 (99.48%)
Number of genes without assigned function	25 (0.52%)
Number of scaffolds	31
Total size of the genome, with gaps (Ns) included.	4,893,875
Scaffolds N50	1,051,362
Average Scaffold length	157,866.93

**Table 4:** Pathway distribution in AZC66.

Level 1	Level 2	Count
09100 Metabolism	09101 Metabolism of carbohydrates	482
	09102 Metabolism of Energy	160
	09103 Metabolism of Lipid	106
	09104 Metabolism of Nucleotide	104
	09105 Metabolism of Amino acid	388
	09106 Metabolism of other amino acids	77
	09107 Biosynthesis and metabolism of Glycan	35
	09108 Metabolism of cofactors and vitamins	208
	09109 Metabolism of terpenoids and polyketides	45
	09110 Biosynthesis of other secondary metabolites	61
	09111 Xenobiotic biodegradation and metabolism	71
09120 Genetic information processing	09121 Transcription	5
	09122 Translation	86
	09123 Folding, sorting, and degradation	51
	09124 Replication and repair	79
09130 Environmental information processing	09131 Membrane transport	136
	09132 Signal transduction	130
09140 Cellular processes	09141 Transport and catabolism	12
	09142 Cell motility	50
	09143 Cell growth and death	22
	09145 Cellular community—prokaryotes	78
09150 Organismal systems	09159 Environmental adaptation	8
09180 Brite hierarchies	09181 Protein families: metabolism	283
	09182 Protein families: genetic information processing	602
	09183 Protein families: signaling and cellular processes	610
09190 Not included in pathway or Brite	09191 Unclassified: metabolism	158
	09192 Unclassified: genetic information processing	29
	09193 Unclassified: signaling and cellular processes	159
	09194 Poorly characterized	125

## Data Availability

Available from the corresponding author upon request.
